# Can the feedback of patient assessments, brief training, or their combination, improve the interpersonal skills of primary care physicians? A systematic review

**DOI:** 10.1186/1472-6963-8-179

**Published:** 2008-08-21

**Authors:** Sudeh Cheraghi-Sohi, Peter Bower

**Affiliations:** 1 National Primary Care Research and Development Centre, University of Manchester, Oxford Road, Manchester, M13 9PL, UK

## Abstract

**Background:**

Improving quality of primary care is a key focus of international health policy. Current quality improvement efforts place a large focus on technical, clinical aspects of quality, but a comprehensive approach to quality improvement should also include interpersonal care. Two methods of improving the quality of interpersonal care in primary care have been proposed. One involves the feedback of patient assessments of interpersonal care to physicians, and the other involves brief training and education programmes. This study therefore reviewed the efficacy of (i) feedback of real patient assessments of interpersonal care skills, (ii) brief training focused on the improvement of interpersonal care (iii) interventions combining both (i) and (ii)

**Methods:**

Systematic review of randomised controlled trials. Three electronic databases were searched (CENTRAL, Medline and Embase) and augmented by searches of the bibliographies of retrieved articles. The quality of studies was appraised and results summarised in narrative form.

**Results:**

Nine studies were included (two patient based feedback studies and seven brief training studies). Of the two feedback studies, one reported a significant positive effect. Only one training study reported a significant positive effect.

**Conclusion:**

There is limited evidence concerning the effects of patient based feedback. There is reasonable evidence that brief training as currently delivered is not effective, although the evidence is not definitive, due to the small number of trials and the variation in the training methods and goals. The lack of effectiveness of these methods may reflect a number of issues, such as differences in the effectiveness of the interventions in experienced practitioners and those in training, the lack of theory linking feedback to behaviour change, failure to provide sufficient training or to use a comprehensive range of behaviour change techniques. Further research into both feedback and brief training interventions is required before these interventions are routinely introduced to improve patient satisfaction with interpersonal care in primary care. The interventions to be tested in future research should consider using insights from the wider literature on communication outside primary care, might benefit from a clearer theoretical basis, and should examine the use of combined brief training and feedback.

## Background

Improving quality of primary care is a key focus of health policy both nationally and internationally [1,2]. Quality improvement can take a number of forms. The approach adopted in the United Kingdom has placed a large focus on the clinical quality of care. Financial incentives in the Quality and Outcomes Framework (QOF) are provided on the basis of achieving certain quality indicators, which at the time of introduction in 2004 included 10 clinical domains of care (76 in total), 56 in organisational areas, four assessing patients' experience, and a number of indicators for additional services [1].

The emphasis on clinical care within the QOF reflects a professional conceptualisation of quality. Patients consistently report that a key priority (alongside technical competence) is the interpersonal skills of their physician [3-5]. The importance of interpersonal care is supported by the fact that most patient complaints centre around issues with doctors' manner, attitude or communication skills [6-8]. Communication skills are central to effective clinical practice such as diagnosis [9], and impact on certain health outcomes [10].

Given the importance of interpersonal skills, the question arises of how best to rectify any deficiencies. Two main methods of quality improvement have been proposed.

Feedback of patient-based surveys has been suggested as a cost-effective quality improvement method [11]. Physicians in both the United Kingdom and the United States are currently remunerated to varying degrees to assess the views of their patient populations. However, the effectiveness of the feedback of patient assessments in improving the quality of interpersonal care is unclear. The controlled trial literature on audit and feedback (defined as being any summary of clinical performance of healthcare over a specified period of time) suggests that it is effective as a strategy to improve professional practice [12,13]. However, such reviews have concentrated on clinical aspects of care such as guideline implementation. It cannot be assumed that feedback of patient assessments will have the same effect as feedback of clinical indicators, as physicians may place greater emphasis on professionally-based audit measures compared with patient assessments [9].

Continuing medical education has been the traditional approach to improve clinical performance, and can range from passive, didactic, large group presentations to highly interactive learning methods, such as workshops, small groups and individualised training sessions. Systematic reviews of the literature have found that medical education can improve clinical performance with the most effective methods being interactive educational meetings, outreach events and strategies that involve multiple educational interventions (e.g. outreach plus reminders) [14-16]. A recent systematic review of the education literature indicated a positive impact on clinical performance when education was coupled with feedback [17].

This study therefore uses systematic review techniques to assess the efficacy of (i) the feedback of patient assessments, (ii) brief training, and (iii) interventions combining both feedback and brief training (i.e. (i) and (ii) together), on the interpersonal skills of primary care physicians.

## Methods

### Inclusion and exclusion criteria

Studies eligible for inclusion were:

1) Randomised controlled trials (RCTs) published in English

2) Based on primary care practitioners and their patients. Primary care practitioners were defined as medical health care professionals providing first contact and on-going care to patients, regardless of the patient's age, gender or presenting problem, and included other relevant specialties such as general internists, family practitioners, paediatricians and obstetricians working in primary care settings. Medical students were excluded from the review. There were no restrictions on age, gender, ethnicity or health condition of patients included in the review.

3) Utilising one of the following interventions:

a) feedback of the assessments of real patients on the interpersonal skills of individual physicians. These assessments (i.e. patient satisfaction scores) were provided to physicians outside the consultation (e.g. written reports);

b) 'brief' (up to one working week in length) training focussed on the improvement of interpersonal care.;

c) interventions combining (a) and (b).

4) Utilising a patient based assessment of change in interpersonal skills as an outcome.

We considered interpersonal care in the broadest sense and included generic interpersonal skills (e.g. listening, providing information) and more specific areas (e.g. shared decision making skills, responding to the patient agenda). We did exclude feedback or training interventions that were specific to a particular disease.

#### Search Strategy

A list of initial search terms and synonyms was formulated by SCS on the basis of the population (primary care physicians and their patients), the interventions (patient based feedback and brief education) and the outcome (patient assessment of interpersonal care). Relevant published studies and reviews e.g[13,18] were reviewed for additional keywords. These searches were combined with the Cochrane Highly Sensitive Search Strategy (HSSS) for randomised controlled trials [19]. The search strategy itself was built by grouping the individual free text and MeSH terms into categories and then combining those components Each search strategy was adapted to each database to ensure the appropriateness of the MeSH terms.

Three electronic databases were searched during April–May of 2007. The primary search was of the CENTRAL register of controlled trials from the Cochrane Library. Subsequently, Medline and Embase searches were conducted limited to the years 2004–2007 in order to capture any articles of relevance that may at that point, due to volume and time constraints, not have been entered into the CENTRAL database. This approach was chosen on the basis of recent published evidence which shows that when searching specifically for RCTs, exhaustive searching of multiple electronic databases is not necessary due to the comprehensive nature of the CENTRAL database [20]. References lists of included articles and of existing published reviews were searched for other relevant articles as well as utilising a citations tracker in order to identify any new relevant literature. The full search strategy is available for the interested reader (see additional file 1).

One reviewer (SCS) applied the inclusion criteria to all the titles and abstracts identified by the electronic searches. Full text copies of all articles judged to be potentially relevant were retrieved for further investigation. Two reviewers (SCS and PB) then independently assessed these articles against the inclusion and exclusion criteria. Any disagreements were resolved via discussion at a series of face to face meetings.

#### Data Abstraction

For each included study, the two reviewers independently performed the data extraction using a modified version of the data collection checklist used by the Effective Practice and Organisation of Care group of the Cochrane Collaboration. Any discrepancies in data extraction or quality assessment were discussed and resolved by consensus. Due to time constraints, it was not possible to contact relevant authors for missing data.

Methodological rigour was assessed by rating individual study criteria as indicators of trial quality [21]. The following eight criteria were assessed:

1) Allocation concealment

2) Power calculation

3) Sample size

4) Follow-up of professionals

5) Baseline comparability

6) Published outcome measurement instrument

7) Protection against contamination

8) Unit of analysis issues

The criteria were assessed in the following way:

##### 1) Allocation concealment

The reviewers assessed this criterion as being 'done' where the unit of allocation was described explicitly and there was some form of centralised randomisation or an adequately concealed method (e.g. sealed opaque envelopes), 'not done' if the allocation was transparent before assignment, or 'not clear' where there was insufficient detail about the allocation method.

##### 2) Power calculation and 3) Sample size

The reviewers assessed power calculations as being 'done' where there was evidence of a power calculation being conducted, 'not clear' if it was not reported and 'not done' if the authors specifically report that the study was under-powered. For sample size, the number of participants reported as being randomised was recorded.

##### 4) Follow-up of professionals

The reviewers assessed this criterion as being 'done' where > = 80% of the professionals randomised had been followed-up, 'not done' if outcome measures had been obtained for less than 80% and 'not clear' if it was not specified within the paper.

##### 5) Baseline comparability

For baseline comparability of intervention and control group participants, we recorded this as being 'done' where the authors had done an analysis of baseline comparability and reported finding no significant differences that may affect the study results, 'not done' if there are significant baseline differences and 'not clear' where no evidence of any analysis of baseline comparability was reported. As statistical significance testing of baseline characteristics is flawed when sample sizes are small, we also report separately any cases that recorded a > 10% differences in measured characteristics at baseline.

##### 6) Published outcome measurement instrument

As we were interested in patient assessments of interpersonal care, it was important that the instruments that study authors had used to measure such assessments were valid and reliable. Although it would have been preferable to investigate the psychometric properties of the measurement tools in more detail, this was not possible due to resource constraints. Therefore, a proxy code was used, concerning whether the instruments were published in a peer reviewed journal or not.

##### 7) Protection against contamination

The reviewers recorded this as 'done' where the physicians were the unit of allocation and 'not done' if patients rather than professionals were randomised.

##### 8) Unit of analysis

Studies which randomize at the level of the clinician or practice but assess the effects on patients need to be analysed correctly, taking into account the difference between the unit of allocation and the unit of analysis. Failure to consider this may lead to inappropriate statistical testing [22]. Appropriate analysis in cluster randomized trials was assessed as 'done' where appropriate adjustment was made for clustering or analysis was conducted at the cluster level, 'not done' when authors were explicit that no adjustment was made, and 'not clear' where there was insufficient detail about adjustment.

For the analysis, study results were analysed by intervention type (i.e. feedback, brief training, and their combination).

## Results

**Figure 1:**
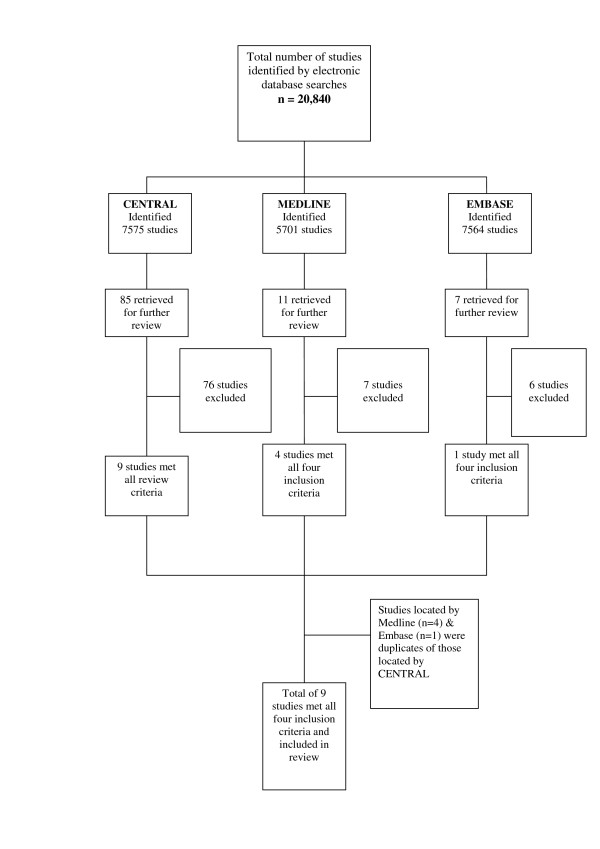
Overview of searching process.

The electronic search identified 20,840 citations (see figure 1). Screening of the titles and abstracts reduced this to 103 after excluding 20,737 ineligible articles. In addition to the electronic search, hand-searching located two further studies. These 105 studies were retrieved and reviewed by both SCS and PB. In total, nine studies were found to meet all four inclusion criteria, comprising two feedback and seven training studies. No studies combining both feedback with training were identified. Two of the included studies were reported across two separate publications respectively [23-26]. For an overview of the characteristics of included studies see additional file 2.

### Participants and settings

General practitioners and their adult patients were the focus of four of the included studies [23,24,27-29], whereas a mixture of primary care physicians were included in the remainder. Three studies [27,30,31] reported on interventions involving trainee physicians such as internal medicine residents, whereas the remaining studies utilised experienced practicing physicians. Five studies were conducted in the United States [25,26,30-33]. The remaining four studies were conducted in the United Kingdom [29]. The Netherlands [23,24] and Australia [27,28]. Further details of physician and patient participants can be found in additional file 3 and additional file 4 respectively.

### Interventions

#### Feedback

Both studies used written feedback as the intervention. The frequency of feedback however did vary, with the first study, providing the intervention five times within a two year period (at three month intervals) [27], whereas in the second study the intervention was only provided once within the fifteen month study period [23,24]. An overview of the feedback interventions is given in additional file 5.

#### Training

Four studies investigated the effects of communication skills training [28,30,32,33] with the remaining studies investigating interventions that target specific interpersonal skills to increase trust [25,26], medical interviewing skills [31] and increasing awareness of the patient agenda [29]. Both individual and group settings were used to deliver training. The control groups in five studies received no training [25,26,28,29,31,33], whereas two studies gave equivalent training except for the specific educational content which was unrelated to interpersonal skills [30,32]. Details relating to the specific content of the training is provided in additional file 6.

### Methodological quality

Of the nine included studies, only one study reported adequate methods of allocation concealment [23,24]. Seven studies reported taking account of the clustered nature of the data in their analyses [23-26,29-33]. Over half the studies performed power calculations [23-27,29,33] and all studies reported following up at least 80% of the professional participants. Sample sizes ranged from 19 to 210 physicians. In terms of baseline comparability of physicians, all studies reported no significant differences in measured characteristics at baseline although in some cases the differences were relatively large. All studies had ensured protection against contamination by randomising the physicians and one study reported specifically asking intervention group physicians not to discuss the intervention with control group physicians [31]. Three of the studies used measurement tools that were not published in peer reviewed journals [28,32,33]. In summary only one study met all the quality criteria [23,24]. A summary of the methodological quality of included studies is given in additional file 7.

#### Outcomes

Meta-analysis was not possible as a minority of studies provided useable data. In addition, there was significant heterogeneity among studies in terms of interventions, which might make a pooled analysis difficult to interpret. Therefore a narrative description of the outcomes is presented.

#### Feedback

In the first of the feedback studies, three groups of trainee physicians were studied over their two year GP vocational training. The two intervention groups both received feedback at five points, with one of the groups also receiving preceptor discussions with their supervisors at two of the intervention points. The control group only received feedback at the first and fifth intervention points. Both intervention groups were found to have significant increases in patient satisfaction compared to control, particularly towards the earlier stages of training, however there were no significant differences between the two intervention groups [27].

In the second study, experienced physicians in the intervention group received an individual written feedback report on patients' evaluations of care whereas the control group received no feedback. The intervention group did not show any significant improvements in patient satisfaction scores, despite physicians reporting making changes in line with feedback [23,24]. Further details of the analysis and results can be found in additional file 8.

#### Training

Only one training study reported a significant positive effect for the communications training intervention [28]. The intervention consisted of two, three hour seminars using both written and oral methods, whereas the control group received no training. The remaining six studies reported that the training interventions had no significant positive effect [25,26,29,31,33]. In one study, the control group actually showed greater improvements in the reported average satisfaction scores than in the intervention group [33]. Further details of the results of training studies can be found in additional file 9.

## Discussion

The aim of this review was to determine the effectiveness of patient based feedback, brief training and their combination on the interpersonal skills of physicians. Only a small number of trials were identified and thus any conclusions about the effectiveness of these interventions is preliminary.

### The effectiveness of patient based feedback

The search identified only two feedback studies, which have been identified previously in a review of instruments and feedback methods for the assessment of physicians using patient surveys [34]. It is unclear from this review whether patient based feedback is an effective quality improvement tool for changing physicians' interpersonal care behaviour. The study involving trainees showed a significant positive effect for patient feedback on patient satisfaction scores [27], whereas the study involving experienced physicians showed no effect [23,24]. A study of feedback excluded from the review (conducted in a hospital setting) also reported a significant positive effect on the interpersonal skills of trainee internal medicine residents [35]. Clinically experienced physicians may have more enduring interpersonal care behaviours that have developed over many years of practice, whereas trainees may be more easily able to adapt their behaviours in line with feedback.

An alternative explanation for the difference in results may relate to the intensity of the feedback, with the study reporting positive effects applying the intervention at five time points (3 months apart) over a two year period [27]. In contrast the study reporting no effect, only gave patient feedback at one time point (3–6 months after the start of the study) within the 15 month study period [23,24].

The use of patient feedback assumes that patients can judge the quality of interpersonal care and that the current assessment technology is capable of capturing patient views. Although doctors and patients have been shown to disagree about what constitutes technical quality of care [36-38], it could be argued that no one is better placed than patients to judge interpersonal performance. There is evidence that patients are able to detect improvements in the quality of the physician-patient interaction [39].

A recent systematic review examined instruments designed to evaluate patients' experiences with individual practicing physicians and whether they are able to provide performance feedback at the individual level [34]. Although many had some evidence of validity, it was generally limited, and it was not clear how well they correlated with other measures of doctor performance. One particular problem with using patient assessment instruments is the so called 'ceiling effect' due to the majority of patients express high levels of satisfaction with care i.e. there is little variation in responses [40-42]. The failure of these instruments to capture negative feedback is another issue that may reduce their effectiveness.

Studies of feedback are unique in that the intervention is very similar to the outcome assessment (i.e. both use patient assessments, although only in the former is the data fed back). If the mere act of measurement (without feedback) were sufficient to change behaviour, then these studies may underestimate the effect of the intervention.

Finally, the authors of the review discussed above [34] found that the aim of feedback was often vague, the exact procedures to be used lacked specificity, and there was a lack of detail about the mechanism by which feedback was expected to lead to improvement, beyond an implicit suggestion of the impact of normative comparisons. The format of feedback may also be important. There was limited detail about the exact form of feedback given in the two studies, although one fed back data on individual questions and nine dimensions of care, with individual data for the GP and reference figures for all GPs [23]. Studies suggest that the style and content of feedback is important [43], and there may be potential in testing different methods of presenting the data and the use of qualitative information from patients to complement quantitative data. Further work on the 'active ingredients' of feedback is clearly required.

### The effectiveness of brief training

Brief training has previously been found to be effective in changing physician behaviour in general [16,44] and reviews focussing specifically on training for interpersonal skills, have also suggested that training can be effective [45]. For example, a Cochrane review of training to improve patient-centredness reported positive effects on a number of outcomes [45]. The difference between the results of the Cochrane review and the current study may reflect differences in outcome measures. The Cochrane review included multiple measures, including process measures of physician behaviour and health outcomes. When restricted to the seven trials using a patient based assessment of interpersonal care skills (the inclusion criteria for the present review) only two of the seven studies in the Cochrane review showed a positive significant effect, a result not substantively different from the results reported here [35,46].

The only positive study was the oldest of all the training studies. This may be due to lower baseline levels of physician interpersonal skills. The medical training undertaken by the participating physicians (whose average age was 41.7 years when the study was undertaken and published in 1987) may have placed less of an emphasis on teaching interpersonal skills as trainees and practitioners were assumed to acquire interpersonal skills incidentally, simply via interacting with patients [47]. Physicians in more recent studies would have undertaken more formal instruction and assessment.

Although the review was restricted to primary care physicians, findings from the wider literature on communication skills for health professionals may be informative in developing more effective interventions. Reviews suggest that effective interventions require combinations of didactic components with practice rehearsal and feedback from peers [48]. Interventions may also need to focus on attitudes that may clash with the interpersonal skills being taught [49]. Another key issue is the length of training. The study used a maximum of one week training as an inclusion criterion, based on discussions with GP colleagues as to what was likely to be feasible in relation to practising GPs. The limited effects of training may simply reflect the limited duration of the interventions, and reflect the paradox that in primary care, effective training may be unfeasible, whereas feasible training may be ineffective [50].

### Limitations of the study

We offer several cautions about the interpretation of these results, over and above caveats concerning the number of identified studies. Firstly, as in all such reviews there is the potential for publication bias. Such bias can lead to an overestimation of an intervention's effect on the outcomes i.e. a false positive [51,52]. Secondly, due to time constraints, we were unable to contact authors for additional information therefore we included only published data. A second consequence of time constraints excluded searching via other means e.g. hand-searching of journals and conference proceedings etc. Thirdly, if studies showing an intervention to be effective are more likely to be published in English, then any summary of only the English language reports retrieved through a database search may result in an overestimate of effectiveness due to a language bias [53,54].

Both of the included studies indicating positive effects did not adjust for clustering. There is a risk of inflating statistical significance when analysing patient level data without adjusting for clustering [55].

The study included trials where the outcome measure was a patient assessment. This criteria was used because interventions that change in patient assessments are likely to be of greater interest to policy makers. However, it should be noted that it may be more appropriate to use a range of assessment technologies (such as process measures of behaviour in the consultation) as well as patient outcomes [48].

The current review was restricted to primary care physicians as they currently provide the majority of care in this setting [56]. Future reviews into these interventions should take into account the potential shift towards increased nurse-led delivery of primary care [57].

#### Implications for research

Although the trials identified in the review were of reasonable quality, their limited number means that confident conclusions about the efficacy of these interventions must await the publication of new studies. A more substantial evidence base is also required to explore the various factors that may affect the efficacy of patient based feedback. Such factors may include the frequency, content and style of feedback and training, and physician and patient characteristics.

The theoretical basis of feedback and training interventions was sometimes unclear. More explicit statements of theory underlying interventions and qualitative research conducted as part of the trials may provide insights into why these interventions succeed or fail.

Thirdly, the effectiveness of patient based feedback in combination with other interventions should be investigated (e.g. the combination of patient based feedback with brief training, or with financial incentives). Financial incentives are known to be effective (external) motivators [58]. This type of arrangement is already utilised in the United States. General practice in the United Kingdom has become accustomed to conducting patient surveys on an annual basis for financial incentives, but the current incentive structure pays physicians primarily on the basis of conducting the survey rather than making changes.

Finally, the cost effectiveness of these interventions need to be assessed. The National Health Service in the United Kingdom has already made a significant financial investment in the process of patient assessments in primary care, and it is critical that this investment can be proven to be a good use of resources compared to other competing priorities.

## Conclusion

There is limited evidence available on the effects of patient based feedback. There is reasonable evidence that brief training as currently delivered is not effective, although the evidence is not definitive, because of the small number of trials and the variation between them in terms of their training methods and goals. Further research into both feedback and brief training is required. The interventions to be tested in future research should consider using insights from the wider literature on communication outside primary care, might benefit from a clearer theoretical basis, and should examine the use of combined brief training and feedback to improve physicians' interpersonal skills.

## Competing interests

PB has been involved in the development of a patient assessment questionnaire which is currently recommended for use by GPs in the United Kingdom as part of their contract. The questionnaire is free to use for NHS staff, but commercial companies selling patient evaluation services are required to pay a license fee. Funds derived from selling the questionnaire in these instances are received by the organization and used to fund research and administration. PB does not gain financially from its use.

## Authors' contributions

The study was conducted as part of a Masters dissertation by SC–S. Both authors developed the idea for the study. SC–S devised the search strategy, reviewed identified articles, extracted data, conducted the analysis and drafted the manuscript. PB assisted with identification of studies and data extraction and aided in the drafting of the manuscript.

## Pre-publication history

The pre-publication history for this paper can be accessed here:



## Supplementary Material

Additional file 1 full CENTRAL search strategy. Click here for file

Additional file 2 Overview of Included Studies. Click here for file

Additional file 3 Population Characteristics – Physicians. Click here for file

Additional file 4 Population Characteristics – Patients. Click here for file

Additional file 5 Intervention – Feedback. Click here for file

Additional file 6 Interventions – Training. Click here for file

Additional file 7 Quality of Included Trials. Click here for file

Additional file 8 Results – feedback. Click here for file

Additional file 9 Results – training. Click here for file
