# Supplementary material for: Can the feedback of patient assessments, brief training, or their combination, improve the interpersonal skills of primary care physicians? A systematic review
Source: BMC Health Serv Res. 2008 Aug 21;8:179. doi: 10.1186/1472-6963-8-179 (PMC2542366; doi:10.1186/1472-6963-8-179)
Supplement: Additional file 1 — full CENTRAL search strategy. [file 1472-6963-8-179-S1.doc]

**CENTRAL SEARCH STRATEGY**

#1 MeSH descriptor Family Practice, this term only

#2 MeSH descriptor Primary Health Care, this term only

#3 MeSH descriptor Community Health Services explode all trees

#4 MeSH descriptor Physicians, Family, this term only

#5 MeSH descriptor Comprehensive Health Care, this term only

#6 MeSH descriptor Patient Care Team, this term only

#7 MeSH descriptor Ambulatory Care, this term only

#8 "shared care":ti,ab

#9 "integrated care":ti,ab

#10 "family practice":ti,ab

#11 "family practitioner":ti,ab

#12 "general practice":ti,ab

#13 "general practitioner":ti,ab

#14 "community care":ti,ab

#15 "family medicine":ti,ab

#16 "family physician":ti,ab

#17 "family physicians":ti,ab

#18 "primary care":ti,ab

#19 "primary health care":ti,ab

#20 "primary healthcare":ti,ab

#21 "family doctor":ti,ab

#22 "family doctors":ti,ab

#23 "primary medical care":ti,ab

#24 "general physician":ti,ab

#25 "general physicians":ti,ab

#26 "general practices":ti,ab

#27 "general practitioners":ti,ab

#28 "primary care practitioners":ti,ab

#29 "primary care practitioner":ti,ab

#30 (community next health):ti,ab

#31 (community next healthcare):ti,ab

#32 "health care":ti,ab

#33 GP:ti,ab

#34 GPs:ti,ab

#35 "primary healthcare team":ti,ab

#36 "primary healthcare teams":ti,ab

#37 "primary medical care":ti,ab

#38 "general internist":ti,ab

#39 "general internists":ti,ab

#40 obstetric*:ti,ab

#41 paediatric*:ti,ab

#42 pediatric*:ti,ab

#43 (#1 OR #2 OR #3 OR #4 OR #5 OR #6 OR #7 OR #8 OR #9 OR #10 OR #11 OR #12 OR #13 OR #14 OR #15 OR #16 OR #17 OR #18 OR #19 OR #20 OR #21 OR #22 OR #23 OR #24 OR #25 OR #26 OR #27 OR #28 OR #29 OR #30 OR #31 OR #32 OR #33 OR #34 OR #35 OR #36 OR #37 OR #38 OR #39 OR #40 OR #41 OR #42)

#44 MeSH descriptor Practice Management, Medical, this term only

#45 MeSH descriptor Quality Assurance, Health Care, this term only

#46 MeSH descriptor Quality Indicators, Health Care, this term only

#47 MeSH descriptor Quality of Health Care explode all trees

#48 MeSH descriptor Education, Professional, this term only

#49 MeSH descriptor Attitude of Health Personnel, this term only

#50 MeSH descriptor Patient Acceptance of Health Care, this term only

#51 MeSH descriptor Cooperative Behavior, this term only

#52 MeSH descriptor Professional-Patient Relations, this term only

#53 MeSH descriptor Professional Competence, this term only

#54 MeSH descriptor Physician's Practice Patterns, this term only

#55 MeSH descriptor Professional Practice, this term only

#56 MeSH descriptor Patient-Centered Care, this term only

#57 MeSH descriptor Education, Medical, Continuing, this term only

#58 MeSH descriptor Professional Role, this term only

#59 MeSH descriptor Physician-Patient Relations, this term only

#60 "quality assurance":ti,ab

#61 "professional behaviour":ti,ab

#62 cpd:ti,ab

#63 "continuing professional development":ti,ab

#64 "patient centered care":ti,ab

#65 "patient centred care":ti,ab

#66 "continuing medical education":ti,ab

#67 (training next program*):ti,ab

#68 (training next intervention*):ti,ab

#69 (training next meeting*):ti,ab

#70 (training next session*):ti,ab

#71 (training next strateg*):ti,ab

#72 (training next workshop*):ti,ab

#73 (education* next program*):ti,ab

#74 (education* next intervention*):ti,ab

#75 (education* next meeting*):ti,ab

#76 (education* next session*):ti,ab

#77 (education* next strateg*):ti,ab

#78 "professional behavior":ti,ab

#79 (#44 OR #45 OR #46 OR #47 OR #48 OR #49 OR #50 OR #51 OR #52 OR #53 OR #54 OR #55 OR #56 OR #57 OR #58 OR #59 OR #60 OR #61 OR #62 OR #63 OR #64 OR #64 OR #66 OR #67 OR #68 OR #69 OR #70 OR #71 OR #72 OR #73 OR #74 OR #75 OR #76 OR #77 OR #78)

#80 MeSH descriptor Feedback explode all trees

#81 MeSH descriptor Interpersonal Relations, this term only

#82 MeSH descriptor Communication explode all trees

#83 MeSH Descriptor Patient Satisfaction explode all trees

#84 (interpersonal next skill*):ti,ab

#85 (consultation next skill*):ti,ab

#86 (communication next skill*):ti,ab

#87 (client next feedback*):ti,ab

#88 (patient next feedback*):ti,ab

#89 (user next feedback*):ti,ab

#90 (consumer next feedback*):ti,ab

#91 (carer next feedback*):ti,ab

#92 (client next evaluation*):ti,ab

#93 (patient* next evaluation*):ti,ab

#94 (user next evaluation*):ti,ab

#95 (consumer next evaluation*):ti,ab

#96 (customer next evaluation*):ti,ab

#97 (carer next evaluation*):ti,ab

#98 (interpersonal next care):ti,ab

#99 feedback:ti,ab

#100 (patient next derived):ti,ab

#101 (patient next mediated):ti,ab

#102 (patient next illicited):ti,ab

#103 (patient next initiated):ti,ab

#104 (#99 OR #100 OR #101 OR #102)

#105 (#98 AND #103)

#106 (feedback near/25 change):ti,ab

#107 (feedback near/25 effect*):ti,ab

#108 (feedback near/25 impact):ti,ab

#109 (feedback near/25 evaluat*):ti,ab

#110 (feedback near/25 compar*):ti,ab

#111 (feedback near/25 modif*):ti,ab

#112 (problem-based next learning):ti,ab

#113 (problem-based next teaching):ti,ab

#114 (problem-based next skill):ti,ab

#115 (problem-based next training):ti,ab

#116 (motivational next interview*):ti,ab

#117 (doctor next patient* next relation*):ti,ab

#118 (doctor next client* next relation*):ti,ab

#119 (physician* next patient* next relation*):ti,ab

#120 (physician* next client* next relation*):ti,ab

#121 (practitioner* next patient* next relation*):ti,ab

#122 (practitioner* next client* next relation*):ti,ab

#123 (doctor next consumer* next relation*):ti,ab

#124 (physician* next consumer* next relation*):ti,ab

#125 (practitioner* next consumer* next relation*):ti,ab

#126 (doctor* next patient* next interaction*):ti,ab

#127 (doctor* next client* next interaction*):ti,ab

#128 (physician* next patient* next interaction*):ti,ab

#129 (physician* next client* next interaction*):ti,ab

#130 (practitioner* next patient* next interaction*):ti,ab

#131 (practitioner* next client* next interaction*):ti,ab

#132 (doctor* next consumer* next interaction*):ti,ab

#133 (physician* next consumer* next interaction*):ti,ab

#134 (practitioner* next consumer* next interaction*):ti,ab

#135 (patient next survey*):ti,ab

#136 (patient next questionnaire*):ti,ab

#137 (#80 OR #81 OR #82 OR #83 OR #84 OR #85 OR #86 OR #87 OR #88 OR #89 OR #90 OR #91 OR #92 OR #93 OR #94 OR #95 OR #96 OR #97 OR #104 OR #105 OR #106 OR #107 OR #108 OR #109 OR #110 OR #111 OR #112 OR #113 OR #114 OR #115 OR #116 OR #117 OR #118 OR #119 OR #120 OR #121 OR #122 OR #123 OR #124 OR #125 OR #126 OR #127 OR #128 OR #129 OR #130 OR #131 OR #132 OR #133 OR #134 OR #135 OR #136)

#138 (#43 AND #79 AND #137)
