# Supplementary material for: Can the feedback of patient assessments, brief training, or their combination, improve the interpersonal skills of primary care physicians? A systematic review
Source: BMC Health Serv Res. 2008 Aug 21;8:179. doi: 10.1186/1472-6963-8-179 (PMC2542366; doi:10.1186/1472-6963-8-179)
Supplement: Additional file 2 — Overview of Included Studies. [file 1472-6963-8-179-S2.doc]

Table 1 Overview of Included Studies

| **Study** | **Country** | **Setting** | **Target Population** | **Sample Size at Baseline** | **Intervention** | **Patient Satisfaction Measure** | **Other Outcomes** |
| --- | --- | --- | --- | --- | --- | --- | --- |
| Greco 2001[27] | Australia | General Practice teaching practices | GP registrars | N=210 | Patient feedback | DISQ | None |
| Wensing 2003 [23], Vingerhoets 2001[24] | The Netherlands | General Practice surgeries | General Practitioners | N=60 | Patient feedback | CEP questionnaire | - Video taped consultations - physician questionnaire data on practice management |
| Evans 1987[28] | Australia | General Practice surgeries | General Practitioners | N=40 | Communication Skills Training | Doctor-Patient Communication Survey | Anxiety |
| Lewis 1991[30] | United States | University Affiliated General Paediatric Practice | Paediatric residents and fellows | N=34 | Communication Skills Training | Parent Medical Interview Satisfaction Scale (PMISS) | *Medical visit processes*   - Consultation content   *Medical visit outcomes*   - Child outcomes:   - health related behaviour & attitudes  - child satisfaction   - Other outcomes:   - parent satisfaction  - physician satisfaction |
| Joos 1996 [32] | United States | University based VA hospital -General Medicine clinics | Staff physicians and General Internists | N=42 | Communication Skills Training | American Board of Internal Medicine Patient Satisfaction Questionnaire | - Patient perception of` reception of information - Medication compliance - Appointment keeping |
| Putnam 1988 [31] | United States | Hospital based medical walk-in clinic | Internal Medicine Residents | N=19 | Medical Interview Skills training | Medical Interview Satisfaction Scale (MISS) | - Patient compliance - Symptom Improvement |
| Middleton 2006 [29] | United Kingdom | General Practice surgeries | General Practice Principals | N=46 | Educational training to increase physician awareness of patient agenda | CSQ | - Duration of consultation - number of problems presented |
| Thom 1999 [25], 2000 [26] | United States | Family Practice | Family Practitioners | N=20 | Educational training to increase physician-patient trust building skills | Consumer Satisfaction Survey | - Trust in physician - Health status - No. of visits to other physicians - Adherence to advice/medication |
| Betz Brown 1999 [33] | United States | HMO Primary Care Clinics | Mixed specialties  (bulk primary care physicians) | N=69 | Communication Skills Training | Art of Medicine Survey | - Clinician self-assessment of physician communication skills |
