# Supplementary material for: Can the feedback of patient assessments, brief training, or their combination, improve the interpersonal skills of primary care physicians? A systematic review
Source: BMC Health Serv Res. 2008 Aug 21;8:179. doi: 10.1186/1472-6963-8-179 (PMC2542366; doi:10.1186/1472-6963-8-179)
Supplement: Additional file 3 — Population Characteristics – Physicians. [file 1472-6963-8-179-S3.doc]

Table 2 Population Characteristics - Physicians

| **Study** | **Target Population** | **Sample Size at Baseline** | **Proportion of eligible physicians that participate** | **Baseline Age**  **(Mean (SD))** | **Baseline sex** | **Experience**  **(yrs)**  **(Mean (SD))** | **Baseline ethnicity** |
| --- | --- | --- | --- | --- | --- | --- | --- |
| Greco 2001[27] | GP registrars | N=210 | Not clear | 29.9  (5.3) | F=60.2%  M=39.8% | All GP registrars at the beginning of their first year of training (median previous experience 2 yrs) | Not clear |
| Wensing 2003 [23], Vingerhoets 2001[24] | General Practitioners | N=60 | 8.57% | 44.3  (5.3) | F=16%  M=84% | 14.5 (6.6) | Not clear |
| Evans 1987[28] | General Practitioners | N=40 | 15.9% | 41.7 | F=0%  M=100% | 16.5yrs (Not clear) | Not clear |
| Lewis 1991[30] | Paediatric residents and fellows | N=34 | 86.1% | 29.3  (Not clear) | F=50%  M=50% | 2.2yrs in Residency | Not clear |
| Joos 1996 [32] | Staff physicians and General Internists | N=42 | 97.7.% | Not clear | F=38.1%  M=62.9% | Training level:  Staff - 33.3%  3^rd^ yr resident – 33.3%  2^nd^ yr resident – 33.3% | Not clear |
| Putnam 1988 [31] | Internal Medicine Residents | N=19 | 100% | 27.6 | F=15.9%  M=84.1% | All in second year of training | Not clear |
| Middleton 2006 [29] | General Practice Principals | N=46 | 8.4% | (Age band & n)  25-29y = 1  30-34y=10  35-39y= 10  40-44y = 16  45-49y = 7  50-54y=0  55-59y=2 | F=28.3%  M=71.7% | Not clear but 63% had a diploma from the Membership of the Royal College of General Physicians (MRCGP) | Not clear but 93.5% UK graduates |
| Thom 1999 [25], 2000 [26] | Family Physicians | N=20 | 30.1% | 47  (12.0) | F=15%  M=85% | 16  (not clear) | 70% white |
| Betz Brown 1999 [33] | Mixed specialties  (bulk primary care physicians) | N=61 | 7.0% | 30-39y = 4  40-49y = 28  50-59y = 25  > 60y =4 | F= 36.1%  M= 63.9% | Not clear | Not clear |
