# Supplementary material for: Can the feedback of patient assessments, brief training, or their combination, improve the interpersonal skills of primary care physicians? A systematic review
Source: BMC Health Serv Res. 2008 Aug 21;8:179. doi: 10.1186/1472-6963-8-179 (PMC2542366; doi:10.1186/1472-6963-8-179)
Supplement: Additional file 4 — Population Characteristics – Patients. [file 1472-6963-8-179-S4.doc]

Table 3 Population Characteristics - Patients

| **Study** | **Target Population** | **Specific Inclusion criteria** | **Proportion of eligible patients that participate** | **Sample size** | **Mean age**  **(SD)** | **Sex** | **Medical condition** | **Other info.**  **(I=intervention group and C= control group)** |
| --- | --- | --- | --- | --- | --- | --- | --- | --- |
| Greco 2001[27] | Consecutive patients | None except willingness to participate | Not clear | 28,156 | 37.5  (16.3) | F=70.4%  M=29.6% | Not clear | 53.1% visiting the GP registrar for the first time |
| Wensing 2003 [23], Vingerhoets 2001[24] | Consecutive patients | - Patients under 18 yrs - Those not able to understand the Dutch Language - Those who were mentally handicapped or terminally ill were excluded. | 67% | 7286 | 49.6  (16.1) | F= 67.7%  M=32.3% | Chronic Illness = 70.9% | Mean no. of visits in past 2 month = 2.30 |
| Evans 1987[28] | Study physician patients | Not clear | Not clear | 400 | 42.09  (Not clear) | F=55%  M=45% | Not clear | Patients of training doctors were older (P<0.01) and spoke a language other than English at home (P<0.001) |
| Lewis 1991[30] | 6-17yr olds accompanied by an adult | Not clear | 80% | 141 | Patient:8.6  Parent:37.8 | Patient:F=43.3%  M=56.7%  Parent: Not clear | Not clear | Previous visits to the physician = 1.6  Ethnicity:  White = 30.5%  Other = 69.5% |
| Joos 1996 [32] | Chronically ill patients | - Attended appointment with study physician in last 9mths - Had another appointment in study period - Were taking oral medication for at least one chronic condition | 22.4% | 348 | 62.3  (3.42) | F=7.2%  M=92.8% | Not clear | - self reported health status:   very poor or poor: I=47% & C=46%  fair: I= 34% & C=35%  good or excellent I=19% & C=19%   - self-rated bother or discomfort from conditions:   None, very little: I=23% & C=24%  Some: I=32% & C=28%  Fair amount: I=25% & C=29%  Great deal: I=20% & C=19%   - Information seeking (Mean KHOS I= 3.36 (SD 0.79) & C= 3.21 (SD 0.75) - Behavioural involvement (Mean KHOS I=2.21 (SD 0.63) and C= 2.04 (SD 0.9) |
| Putnam 1988 [31] | Patients attending walk-in clinic for non-emergency problems | - Literate - Home telephone available - Not previously seen physician | 58.3% | 268 | 32.6  (Not clear) | F =67.2%  M=32.8% | Chronic Illness =28.0% | Mean Education (yrs)=11.8yrs  Ethnicity:  White = 41.8%  Other = 58.2% |
| Middleton 2006 [29] | Study physician patients | None except willingness to participate in study | 88% | 857 | Not clear | Not clear | Not clear | Not clear |
| Thom 1999 [25], 2000 [26] | Consecutive patients | Excluded:   - Those < 18yrs old - Unable to complete questionnaire - Those in acute distress - New patients to physician | 73.8% | 414 | 47.25  (16.1) | F = 61.8%  M =38.2 % | One or more chronic conditions = 51.4% | Median length of relationship  = 26.5 mths |
| Betz Brown 1999 [33] | Not clear | Not clear | Not clear | Not clear | Not clear | Not clear | Not clear | Not clear |
