# Supplementary material for: Can the feedback of patient assessments, brief training, or their combination, improve the interpersonal skills of primary care physicians? A systematic review
Source: BMC Health Serv Res. 2008 Aug 21;8:179. doi: 10.1186/1472-6963-8-179 (PMC2542366; doi:10.1186/1472-6963-8-179)
Supplement: Additional file 5 — Intervention – Feedback. [file 1472-6963-8-179-S5.doc]

Table 4 Intervention - Feedback

| **Study** | **Feedback Description** | **Control Description** |
| --- | --- | --- |
|
| Greco 2001[27] - 1 | *Intervention 1 :* ‘Serial feedback’ group  Type: Feedback scores from patient surveys  Theoretical basis Not clear  Specific content: Overall DISQ score or Interpersonal Skills Index (ISI) plus per item score  Frequency: 5 times  Duration: Not clear  Guidance: None    Format: Written  Deliverer: Not clear  Timing of feedback: Feedback received towards end of B1 and G2 terms of the Royal Australian College of General Practitioners’ (RACGP) Training Program as well as during their B2, A2 and G1 terms. Therefore approx. at 3, 6, 12, 15 and 18 months respectively after the start of the study. | Control group: Allowed for a Pre- and post-test comparison  Type: Feedback scores from patient surveys  Theoretical basis Not clear  Specific content: Overall DISQ score or Interpersonal Skills Index (ISI) plus per item score  Frequency: 2 times  Duration: Not clear  Guidance: None    Format: Written  Deliverer: Not clear  Timing of feedback: Feedback received towards end of B1 and G2 terms of the Royal Australian College of General Practitioners’ (RACGP) Training Program i.e. approx. at 3 months and 18 months respectively after the start of the study. |
| Greco 2001[27] - 2 | *Intervention 2:* ‘GPS discussion’ group (preceptor feedback)  Type: Feedback scores from patient surveys  Theoretical basis: Not clear    Specific content: Overall DISQ score or Interpersonal Skills Index (ISI) plus per item score  Frequency: 5 times  Guidance: Discussion involved preceptor feedback from their supervisors at 2 out of the 5 feedback points  Format: Written    Deliverer: GP supervisor (GPS)  Timing of feedback: Feedback received towards end of B1 and G2 terms of the Royal Australian College of General Practitioners’ (RACGP) Training Program as well as during their B2, A2 and G1 terms. In addition Registrars had supplementary preceptor feedback from their GPSs during the B2 and A2 terms. Therefore approx. at 3, 6, 12, 15 and 18 months respectively after the start of the study. | Type: Feedback scores from patient surveys    Theoretical basis Not clear  Specific content: Overall DISQ score or Interpersonal Skills Index (ISI) plus per item score  Frequency: 2 times  Duration: Clear  Guidance: None    Format: Written  Deliverer: Not clear  Timing of feedback: Feedback received towards end of B1 and G2 terms of the 2^nd^ year of the Royal Australian College of General Practitioners’ (RACGP) Training Program i.e. approx. at 3 months and 18 months respectively. |
| Wensing 2003 [23], Vingerhoets 2001[24] | Type: Feedback scores from patient surveys  Theoretical basis: Not clear but some discussion around provider sensitivity to patient dissatisfaction and therefore feedback of patient satisfaction scores thought to result in undertaking appropriate activities for improvement of such scores.  Specific content: 15 page individual written feedback report. Report contained figures for each question and aggregated scores for the nine dimensions of care in the CEP. Reference figures related to patients from all study GPs added to allow for comparison of personal performance amongst peers, as well as an abstract of a systematic review of studies of patients’ evaluations of care.  Frequency: Information provided once.  Duration: Average of 50mins reading the report  Guidance: Short manual giving guidance on how to use the results, including discussions with colleagues, detailed follow-up surveys among patients and the establishment of a patient panel.  Format: Written  Deliverer: Not clear  Timing of feedback: Intervention provided 3-6 months after the start of the study. | Type: No feedback provided. Feedback given only after the post-intervention measurements had been taken i.e. approx. 15months after start of study for information purposes only. |
|  |  |  |
