# Supplementary material for: Can the feedback of patient assessments, brief training, or their combination, improve the interpersonal skills of primary care physicians? A systematic review
Source: BMC Health Serv Res. 2008 Aug 21;8:179. doi: 10.1186/1472-6963-8-179 (PMC2542366; doi:10.1186/1472-6963-8-179)
Supplement: Additional file 6 — Interventions – Training. [file 1472-6963-8-179-S6.doc]

Table 5 Interventions - Training

| **Study** | **Training Description** | **Control Description** |
| --- | --- | --- |
|
| Evans 1987[28] | Intervention type: Communication skills training  Theoretical Basis: Not clear  Specific content: Physicians given booklet, then one hour lecture on patient satisfaction. Remaining time spent in discussion within the group on the techniques of communicating with patients. The nature of second session- not clear.  Intervention site: Not clear  Frequency & Duration: 2 x 3 hr seminars  Total Duration: 6 hrs  Delivery format: Written & oral - lectures by first author and discussion.  Mode of Training: Groups (n=10)  Deliverer: First author | No training given |
| Lewis 1991[30] | Intervention type: Communication skills training  Theoretical Basis: Not clear  Specific content: Training session involved viewing of 15min videotape presentation presenting research evidence on communication issues, vignettes demonstrating communication skills (e.g. building rapport, checking on understanding etc.) and review of research articles. Boosters sessions content not clear.  Intervention site: Not clear  Frequency & Duration: 1 main 1hr session plus three written boosters  Total Duration: 1hr plus three boosters of unspecified length.  Delivery format: Video, written and oral - reflective learning.  Mode of Training: Not clear  Deliverer: Not clear | Intervention type: Educational febrile infants training of the same mode, format, frequency and duration. |
| Joos 1996 [32] | Intervention type: Communication skills training  Theoretical Basis: Not clear  Specific content: Program designed to enhance their ability to elicit, identify and respond effectively to patient requests.  Session 1: Importance of eliciting patient agenda, negotiating agenda, identifying attributions & expectations, relationship building and using clinical tool (Patient Requests for Services Questionnaire – elicits patient preferences for particular services or assistance required).  Session 2: Review experience with clinical tool and focus on aiding patients follow recommendations.  Session 3: Involved practice & feedback of skills using simulated patients  Intervention site: Work place (General Medicine Clinic)  Frequency & Duration: 3 x 90min sessions at two week intervals  Total Duration: 270mins (4.5hrs)  Delivery format: Readings, lecture, discussion, review of videotapes and role playing plus practice with the clinical tool between training sessions  Mode of Training: Individual and group  Deliverer: Not clear | Intervention Type: Educational medical decision making training of same mode, format, frequency and duration. |
| Putnam 1988 [31] | Intervention type: Medical Interview Skills training    Theoretical Basis: Not clear but biopsychosocial perspective highlighting open and thorough exchange of information, based in empirical studies of patient exposition and physician explanation.  Specific content: Group sessions: active listening & giving thorough information  Individual session: reviewed tape recordings of patient interviews focussing on listening and explanations. Techniques for giving information also discussed.  Intervention site: Not clear  Frequency & Duration: 1-2 group sessions plus 5-6 individual sessions.  Total Duration: Average of 3.7hrs, of which 62% was spent in individual sessions  Delivery mode: Written (manual) and oral (role-playing).  Mode of Training: Individual and group, plus short written manual.  Deliverer: First author – internist with 10yrs experience in medical interviewing. | No training given - Although after the study, the residents in the control groups were offered the training. |
| Middleton 2006 [29] | Intervention type: Educational training to increase physician awareness of patient agenda  Theoretical Basis: Not clear but model focuses on identifying the layers of the patient’s agenda (ideas, concerns, expectations and reasoning) the doctors explicitly reflecting on their own agenda (care of presented and continuing problems, risk factors and practice and continuing factors) and negotiation of action with the patient.  Specific content: Not clear but model focuses on identifying the layers of the patient’s agenda (ideas, concerns, expectations and reasoning) the doctors explicitly reflecting on their own agenda (care of presented and continuing problems, risk factors and practice and continuing factors) and negotiation of action with the patient. Physicians practiced model on simulated patients and given feedback on performance.  Intervention site: Not clear  Frequency & Duration: One ‘day’  Total Duration: One ‘day’  Delivery format: Not clear but assumes face to face as intervention used workshop format  Mode of Training: Not clear but assume group due to workshop format  Deliverer: First author | No training given - Although after the study, the physicians in the control groups were offered the training after the study was completed. |
| Thom 1999 [25], 2000 [26] | Intervention type: Educational training to increase physician-patient trust building skills  Theoretical Basis: Not clear  Specific content: Teaching around 7 behaviours that increase patient trust (thoroughly evaluating problems, understanding of patients’ experience, expressing caring, providing appropriate and effective treatment, communicating clearly and completely, building partnership and demonstrating honesty and respect.  Intervention site: Not clear  Frequency & Duration: 1 x 7hr session  Total Duration: 7hrs  Delivery format: Workshop, Problem-based learning techniques, didactic presentations, videotaped encounters with patients, group discussion, role-playing  Mode of Training: Group  Deliverer: 2 physicians from the Division of Family and Community Medicine and a sociologist | No training given |
| Betz Brown 1999 [33] | Intervention type: Communication Skills Training  Theoretical Basis: Not clear  Specific content: 1^st^ workshop – focussed skills for building effective relationships with patients, including listening actively; responding to patients’ feelings; and communicating concern, understanding, and respect.  2^nd^ workshop - focused on skills for successful negotiation, particularly in situations of disagreement (for example, when a patient requests narcotics for chronic pain and the clinician does not think a prescription is appropriate).  Intervention site: Not clear  Frequency & Duration: 2 x 4hr workshops plus 2hrs homework    Total Duration: 10hrs  Delivery format: Oral and written (role-playing)  Mode of Training: Individual and Group  Deliverer: Two physicians who were experienced teachers and experts in clinician–  patient communication. | No training given but offered training at a later date. |
