# Supplementary material for: Can the feedback of patient assessments, brief training, or their combination, improve the interpersonal skills of primary care physicians? A systematic review
Source: BMC Health Serv Res. 2008 Aug 21;8:179. doi: 10.1186/1472-6963-8-179 (PMC2542366; doi:10.1186/1472-6963-8-179)
Supplement: Additional file 7 — Quality of Included Trials. [file 1472-6963-8-179-S7.doc]

Table 6 Quality of Included Trials

| **Study** | **Conceal-ment** | **Power Calculation** | **Sample Size (n)** | **80% Follow-up Prof** | **Baseline Comparability** | **Protection against contamination** | **Adjustment for clustering** | **Measurement tool published?**  **(Yes/No)** |
| --- | --- | --- | --- | --- | --- | --- | --- | --- |
| Greco 2001[27] | Not Clear | Done | 210 | Done | *Physicians* – Done - Reported as comparable on all 5 characteristics. (However, > 10%­Differences on gender, source of entry & practice type)  *Patients* – Reported incomparable on characteristics of age, gender and level of consultation difficulty (P<0.05). | Done | Not clear | Yes – DISQ |
| Wensing 2003 [23], Vingerhoets 2001[24] | Done | Done | 60 | Done | *Physicians* – Done - Reported as comparable on all 4 characteristics  *Patients* – Reported as comparable on 5//6 characteristics except pre-intervention group had significantly higher freq. of attendance (p=0.0001). | Done | Done | Yes – CEP |
| Evans 1987[28] | Not Clear | Not clear | 40 | Done | *Physicians* – Done- Reported as comparable on all 5 characteristics  *Patients*: reported as homogeneous on most variables measured except age and ethnicity P<0.01) | Done | Not clear | No – Doctor-patient communication survey |
| Lewis 1991[30] | Not Clear | Not Clear | 34 | Done | *Physicians* – Done - Reported as comparable on all six characteristics (p<0.2)  *Patients*: Reported as comparable on all 19 characteristics except 9% diff in those accompanied by father p<0.1 | Done | Done | Yes – Parent Medical Interview Satisfaction scale |
| Joos 1996 [32] | Not Clear | Not Clear | 42 | Done | *Physicians* – Done - Reported as comparable on all 7 characteristics (however 15% diff in sex)  *Patients* - Reported as comparable on all 7 characteristics | Done | Done | No – American Board of Internal Medicine Patient Satisfaction Questionnaire |
| Putnam 1988 [31] | Not Clear | Not clear | 19 | Done | *Physicians* – Done -Reported as comparable on both characteristics  *Patients*: Reported as comparable on all 6 characteristics (however 19.6% diff in ethnicity) | Done | Done | Yes - MISS |
| Middleton 2006 [29] | Not clear | Done | 46 | Done | *Physicians*: Done - No reported diff in comparability (however > 10% diff on UK graduate, urban and semi-rural/rural practices)  *Patients*: Not clear | Done | Done | Yes – CSQ |
| Thom 1999 [25], 2000 [26] | Not Clear | Done | 20 | Done | *Physicians* – Done –No reported diff in comparability (however 20% diff in those in group practice)  *Patients* – No reported diff in comparability on all 7 characteristics | Done | Done | Yes – Consumer Satisfaction Survey |
| Betz Brown 1999 [33] | Not Clear | Done | 61 | Done | *Physicians* – Done -Reported comparable both characteristics ( sex and age )  *Patients* – Not clear | Done | Done | No -Art of Medicine Survey |
