# Supplementary material for: Can the feedback of patient assessments, brief training, or their combination, improve the interpersonal skills of primary care physicians? A systematic review
Source: BMC Health Serv Res. 2008 Aug 21;8:179. doi: 10.1186/1472-6963-8-179 (PMC2542366; doi:10.1186/1472-6963-8-179)
Supplement: Additional file 8 — Results – feedback. [file 1472-6963-8-179-S8.doc]

Table 7 Results - feedback

| **Study** | **Analysis** | **Results** |
| --- | --- | --- |
| Greco 2001[27] | Multivariate analyses examining three programmatic effects were examined:  (1) ‘training program effect’ measured whether physicians skills had improved by undertaking the educational activities,  (2) the ‘intervention effect’ provided an indication of the degree that physicians skills had improved as a result of the two interventions  (3) the ‘stage effect’ examined the impact of receiving feedback at various stages throughout the physicians training. | (1) training program effect’ – Only one DISQ item ‘patient confidence in the physician’s ability’ showed significant improvement from pre-to post-test (*P*=0.01)  (2) the ‘intervention effect’ – No significant differences were found between intervention groups for all second order variables(*P*>0.05). Conversely, both intervention groups had significantly higher least mean square scores (LSMs). No sig. differences were found between the intervention and control groups, nor between the two intervention groups, for 3^rd^ order outcome variables(*P*>0.05).  (3) the ‘stage effect’ – For between stage effects, the GPS discussion group had significantly higher LSMs than the serial feedback group for 7 DISQ items and the ISI score. For within stage effects, all DISQ items showed significant increases in LSM scores for the overall training period.  Summary  Analysis indicated that there was a negligible increase in the LSMs scores for the control group, however both the intervention groups showed significant improvements in their LSM scores (interpersonal skills scores) during the training program. Effects were greatest at the earlier stages of training.  However, feedback had inconsistent effects on interpersonal skills as measured by the DISQ. 5 variables out of 12, which were under the direct control of the physicians were more resistant to change given the interventions. |
| Wensing 2003 [23], Vingerhoets 2001[24] | An aggregated or mean score for each dimension of the CEP was calculated. In addition a series of multilevel regression analyses for each dimension using a GP factor was performed. | Model A: Focussed on pre- and post-intervention difference measurements and found only one significantly different effect of group allocation (intervention vs. control): evaluations of ‘medical care’ improved in the control group by 0.09 but in the intervention group by only 0.01 (p=0.035).  Model B: Model used post-intervention scores as dependent variables and found that the proportion of explained variance in the patient evaluation scores determined solely by the GP was highest for CEP dimensions of ‘premises’ and ‘organisation of appointments.  Model C: Included group allocation as a potential predictor and revealed that patients’ evaluations of ‘continuity’ were less positive in the intervention group than in the control group (p= 0.0236).  Model D: Model included pre-intervention scores as predictors and effect was slightlymodified but slightly moderated at p=0.0479.  Model E: Model explored whether patients’ evaluations changed when GPs reported taking actions in line with patient feedback showed no significant effect.  Summary  Feedback had little influence on patient evaluations of physicians even when physicians had reported making changes in accordance with the feedback. |
