# Supplementary material for: Can the feedback of patient assessments, brief training, or their combination, improve the interpersonal skills of primary care physicians? A systematic review
Source: BMC Health Serv Res. 2008 Aug 21;8:179. doi: 10.1186/1472-6963-8-179 (PMC2542366; doi:10.1186/1472-6963-8-179)
Supplement: Additional file 9 — Results – training. [file 1472-6963-8-179-S9.doc]

Table 8 Results - training

| **Study** | **Analysis** | **Results** |
| --- | --- | --- |
| Evans 1987[28] | Independent group t-tests were used where group variances were significantly different, in which cases Fisher-Behren’s t’ was used. | Intervention group: mean =99.3, SD=3.9  Control group: mean = 83.9,SD=10.5, t=7.5., d.f. =398 P<0.001  Overall patient satisfaction was higher in the patients of the intervention group than the patients in the control group indicating that training in this case was effective. |
| Lewis 1991[30] | Data were analysed using mixed effects ANOVA.  Parent gender and experimental status were included as fixed effects, and physician was included as a random effect. | Intervention group: mean = 1.56, SD= 0.43  Control group: mean = 1.63, SD=0.41  Reported parent satisfaction did not vary between intervention and control groups. |
| Joos 1996 [32] | Unit of analysis – physicians for both analytical approaches namely:  1. Generalised estimating equations (GEE)  2. General linear model analysis of variance.  The model included terms for treatment effect, physician effect and a covariate consisting of pre-intervention values of elicitation of concerns. | Satisfaction scores were high for the majority of physicians.  Patient satisfaction before intervention: Intervention group=4.40, SD=0.23 and control group=4.37,SD=0.19  Patient satisfaction after intervention: Intervention group=4.44, SD=0.22 and control group=4.36,SD=0.22  Satisfaction scores did not change significantly after the intervention in either the intervention or control group (P>0.20). |
| Putnam 1988 [31] | Mean values for all variables were calculated and tests of significance were performed on mean differences to test the probability that the differences were equal to zero. | Satisfaction was analysed in terms of cognitive and affective satisfaction  Cognitive satisfaction: Intervention group difference before and after training =-0.1, SE of difference = 0.2. Control group difference before and after training =-0.4, SE of difference = 0.2.  Affective satisfaction: Intervention group difference before and after training =-0.1, SE of difference = 0.1. Control group difference before and after training =-0.2, SE of difference = 0.2.  There were no significant differences in the satisfaction scores. |
| Middleton 2006 [29] | Multilevel models were fitted to account for any clustering effects as patient were clustered within doctors | Patients in the intervention group reported greater satisfaction with the depth of the physician patient relationship, which was increased by the use of the patient agenda form (3.0 (0.5 to 5.6); P=0.02).  General satisfaction: reference group - Mean= 83.6 (95% CI = 81.5 to 85.8)  Change in means (95%CI) (reference group –intervention group) :  No education plus agenda form = 1.4 (-1.1to 3.8)  Education plus no agenda form = -0.3(-3.2 to 2.7)  Education plus agenda form = 0.1 (-2.9 to 3.0) |
| Thom 1999 [25], 2000 [26] | Effect of the intervention on outcomes at 6 mths was tested using ANOVA. The specific model included a study phase variable (before/after intervention), an intervention group variable (intervention/control) and dummy variables for each pair of physicians and two-way interaction terms. | Mean patient satisfaction scores were as follows:  Pre-intervention:  a)Intervention group – 78.2  b)Control group – 79.1  Post-intervention  c) Intervention group – 76.1  d) Control group -76.5  Net difference (c-d) – (a-b)= 0.5  Overall there was no evidence for a difference between the intervention and control groups |
| Betz Brown 1999 [33] | Generalised estimating equations represented a two-level model to account for the influence of within-participant (intra-clinician) correlation on outcome scores.  The model also controlled for baseline scores on the Art of Medicine Survey and for secular changes in scores. Specifically the model was specified to include the following dichotomous terms: Group (control vs. intervention), period (pre-intervention vs. post-intervention) and group x period. The dependent variable was the average of all non-missing Art of Medicine ratings. | Overall, satisfaction with the physician’s service by mean scores:  Intervention group:  Pre-intervention score – 8.04  Post-intervention score – 8.09  Change between scores = 0.05  Control group:  Pre-intervention score – 8.14  Post-intervention score – 8.18  Change between scores = 0.04  The mean score on the Art of Medicine survey improved more in the control group than in the intervention group: mean and 95% CI = 0.072 [-0.010 to 0.154] vs. 0.030 [-0.060 to 0.120]. The difference in changes was 0.042 [-0.080 to 0.164].  In summary, exposure to the communications skills program did not affect changes in the patient satisfaction scores. |
